# Depressive disorders and intravenous drug use in chemsex context

**DOI:** 10.1192/j.eurpsy.2024.668

**Published:** 2024-08-27

**Authors:** J. Curto Ramos, A. Rodríguez Laguna, P. Barrio, L. Ibarguchi, A. García, I. Azqueta, H. Dolengevich Segal

**Affiliations:** ^1^Department of Psychiatry, Clinical Psychology and Mental Health, La Paz University Hospital; ^2^ Apoyo Positivo; ^3^Dual Disorders Program. Department of Psychiatry, Henares University Hospital, Madrid, Spain

## Abstract

**Introduction:**

Several studies have called atention to the mental health disorders associated with chemsex -the intentional use of drugs before or during sexual intercourse GBMSM (gay, bisexual and men who have sex with men) population-. Sexualized intravenous drug use is also known as slam or slamsex. There are few studies that analyze the mental health differences between intravenous drug users compared to non-intravenous drug users in chemsex context.

**Objectives:**

We aim to describe the mental health outcomes including current and past depressive disorders diagnosis in a sample of users with sexualized drug use (chemsex) attended by the non-governmental organization Apoyo Positivo in the program “Sex, Drugs and You” and to compare the differences of current and previous diagnosis of depressive disorders between intravenous drug users compared to non-intravenous drug users.

**Methods:**

A cross-sectional descriptive analysis of a sample of users attended by the non-govenrmental organization Apoyo Positivo in the program “Sex, Drugs and You” between 2016-2019 was performed.

**Results:**

We included 217 participants. Current or past diagnosis of depression was found in 137 participants. Depressive disorders were significantly higher in the intravenous drug use group compared to the non-intravenous drug use group (p<0.05).

**Conclusions:**

Our study reports high levels of depression in chemsex users. The participants in our sample who engaged in intravenous drug use presented a higher frequency of depressive disorders than non intravenous drug use participants. Further studies analyzing the relationship between chemsex, slamsex and depresssion are needed. A multidisciplinary team is necessary to address chemsex and provide care and mental health treatment to chemsex users.

**Disclosure of Interest:**

None Declared

